# Emotional response to a therapeutic technique: The social Broad Minded Affective Coping

**DOI:** 10.1111/papt.12095

**Published:** 2016-04-20

**Authors:** Natasha Holden, James Kelly, Mary Welford, Peter J. Taylor

**Affiliations:** ^1^ Psychosis Research UnitGreater Manchester West NHS Mental Health Foundation TrustPrestwichUK; ^2^ Lancashire Care NHS Foundation TrustEarly Intervention ServiceAccringtonUK; ^3^ Compassion in MindBudeCornwallUK; ^4^ Institute of Psychology, Health & SocietyUniversity of LiverpoolUK

**Keywords:** compassion, imagery, emotion, self‐attacking, threat

## Abstract

**Objectives:**

It has been suggested that savouring positive memories can generate positive emotions. Increasing positive emotion can have a range of benefits including reducing attention to and experiences of threat. This study investigated individuals' emotional reactions to a guided mental imagery task focussing on positive social memory called the ‘social Broad Minded Affective Coping (BMAC)’ technique. The study examined possible predictors of individuals’ responses to this intervention.

**Method:**

An internet‐based, within‐group, repeated‐measures design was used. One hundred and twenty‐three participants completed self‐report measures of self‐attacking and social safeness/pleasure. They were then guided through the social BMAC. Participants completed state measures of positive and negative affect and social safeness/pleasure before and after the intervention. Forty‐nine participants took part in a 2‐week follow‐up.

**Results:**

It was found that safe/warm positive affect, relaxed positive affect and feelings of social safeness increased following the social BMAC, whilst negative affect decreased. In addition, it was found that people scoring higher on inadequate self‐attacking benefited most from this intervention. Changes in affect were not maintained at the 2‐week follow‐up.

**Conclusion:**

The results provide preliminary support for the efficacy of the social BMAC in activating specific types of mood (those associated with safeness rather than drive/reward). This task has potential as part of therapeutic interventions directed at clinical groups, but further evaluation is needed.

**Practitioner points:**

- The social Broad Minded Affective Coping (BMAC) was related to improvements in forms of positive affect linked to the affiliative system.
- This task may be helpful in inducing these positive mood states within therapy.
- Further evaluation comparing the BMAC to a control task is needed.
- Individuals with a greater fear of compassion or more hated‐self‐criticism may gain less from the task, although effects were small.

## Background

Broad Minded Affective Coping (BMAC; Tarrier, 2010) is an intervention that aims to elicit positive affect through the use of mental imagery of a positive memory. The BMAC has been used as a therapeutic technique in a Cognitive Behavioural Suicide Prevention for Psychosis trial with the aim of reducing threat within therapy sessions, bringing about change through building positive schemas, increasing sense of agency and reducing retrieval bias for negative memories (CBSPp; Tarrier & Gooding, 2009; Tarrier *et al*., 2013, 2014). This technique has been found to increase hope and happiness in individuals with psychosis (Johnson, Gooding, Wood, Fair, & Tarrier, 2012). Anecdotal evidence has found the BMAC to be clinically feasible and acceptable (Tarrier, 2010). This study aimed to investigate individuals' emotional reactions to the mental imagery of a positive social memory using the BMAC technique. A secondary aim was to examine possible predictors of individuals' responses to this intervention. It was expected that the results would help to indicate when the social BMAC may be used most optimally in clinical settings, and what factors may contraindicate its use.

It has been well documented that mental imagery can elicit strong emotional responses (Holmes & Mathews, 2010; Holmes, Mathews, Mackintosh, & Dalgleish, 2008). The BMAC technique uses mental imagery to help a person to savour a positive memory with the aim of eliciting positive emotions. Savouring is thought to generate positive emotions through attending to a positive event or feelings about a positive event from either the past, the present, or the hypothetical future (Bryant & Veroff, 2007). The BMAC aims to bring an individual's attention to the sensory components and the emotions associated with the positive memory, as well as eliciting, elaborating and processing personal meaning held by the individual that may run counter to more negative beliefs. Increasing positive emotion can have a range of benefits. Positive emotions are associated with increased mental wellbeing, better physical health and occupational success and thought to increase access to more psychological resources, broaden potential behavioural options and reduce attention to, and experiences of, threat (Fredrickson, 2001; Lyubomirsky, King, & Diener, 2005).

The work of Depue and Morrone‐Strupinsky (2005) encourages us to look beyond the unitary positive affect construct and to focus on distinct brain systems when considering threat regulation. Through examining psychometric and neurobehavioural evidence, they suggest that positive emotions actually comprise of at least two distinct brain systems; a dopamine seeking drive‐based reward system and an oxytocin‐opiate system of contentment, soothing and safeness. The former is linked to achievement‐based emotions such as excitement whilst the latter is tied to feelings of contentment, safeness, and warmth. These can be distinguished from negative, threat‐based emotions such as fear, anxiety, anger, and disgust, which are important in that they alert us to danger, and down‐regulate motivations for exploration and pro‐social behaviours (Gilbert, 2005).

Gilbert (2005) suggested that the reward, soothing and threat systems form a tripartite model of affect regulation, each with the potential to down‐regulate the other. The system of contentment, soothing and safeness, or the affiliative system, is the main regulator of the threat system. This theory is supported by Kelly, Zuroff, Leybman, and Gilbert (2012) who found that social safeness is an emotional response to affiliation which may then protect against psychosocial suffering. If this is the case, then savouring positive affiliative emotions may help to regulate threat.

A well‐balanced integration of threat‐based and positive‐based emotions allows us to negotiate a complex world of acquiring rewards, gaining and giving care and also remaining safe from threat (Gilbert, 2012; Le Doux, 1998; Panksepp, 1998). An inability to down‐regulate threat‐based emotions can have numerous negative consequences for an individual's mental health (Bowlby, 1969; Gerhardt, 2004). Garland *et al*. (2010) suggest the down regulation of threat occurs through the stimulation of ‘positive emotions’. In their review of evidence from behavioural and brain sciences research, they argue that positive and negative emotional states tend to be self‐perpetuating spirals, engendering more of the same. Hence, feelings of shame may trigger social avoidance and emotion‐consistent appraisals which further maintain these feelings. They suggest that an upward spiral of positive emotions can counteract the downward spiral of threat‐based emotions that characterize psychopathology. Garland *et al*. (2010) argue that these positive and negative emotional states are incompatible, so that stimulated feelings of contentment and warmth should be able to replace previous threat‐based emotions (fear, shame). Furthermore, they suggest that repeated activation of positive emotions may result in changes in brain function and structure that confer long‐term resilience to negative emotions and ultimately, emotional difficulties. For example, positive mood has been linked to activation of reward circuits involving dopamine release in several brain areas (nucleus accumbens, striatum, cortical and limbic areas; Mitchell & Phillips, 2007), and the potential plasticity of such circuits has been suggested by observations of change following long‐term use of substances which activate these systems (Garland *et al*., 2010). Garland *et al*. (2010) also suggest that the savouring of pleasant life events is one way that this could be achieved. Savouring positive emotions through the use of mental imagery would therefore be expected to boost threat regulation.

Compassion focussed imagery has been found to increase positive affect (Jacob *et al*., 2011; Rockliff *et al*., 2011), decrease negative affect (Jacob *et al*., 2011; Lincoln, Hohenhaus, & Hartmann, 2013) and reduce feelings of shame (Kelly, Zuroff, & Shapira, 2009). However, it has been found that individuals who are more self‐critical find it harder to self‐soothe when receiving compassionate imagery (Rockliff, Gilbert, McEwan, Lightman, & Glover, 2008), are more resistant to positive emotions associated with compassion, and find it more difficult to receive compassionate emotions (Duarte, McEwan, Barnes, Gilbert, & Maratos, 2015; Rockliff *et al*., 2011). It has been proposed that self‐attacking can take different forms with different functions (Gilbert, Clarke, Hempel, Miles, & Irons, 2004). The function of inadequate self‐attacking is thought to be for self‐correction, whereas the function of hated self‐attacking is thought to be for self‐punishment.

It is thought that experiencing care and soothing from a parent stimulates oxytocin and endorphins and creates calming feelings that can reduce threat (Carter, 1998). Drawing from attachment theory (Bowlby, 1969), if a parent is able to soothe their child when the child becomes distressed, they will stimulate pathways in their child's brain that will enable the child to self‐soothe later in life (Gilbert, 2009). Individuals who have received this care would be expected to have a well‐developed affiliative system which could regulate threat. However, if an individual has experienced harsh and punishing environments, they may have developed a threat system that dampens down affiliative emotions in order to prevent external attack and will therefore be fearful of compassion (Gilbert, 2000). This can be seen as an adaptive strategy as the individuals affect system is adjusting to an environment where they expect harsher treatment from others. However, operating this way could have longer term negative consequences.

In everyday life people regulate their levels of threat through affiliative interactions and self‐soothing. However, as discussed, some people find it more difficult to self‐soothe than others, particularly those with higher levels of shame and self‐criticism (Gilbert & Proctor, 2006). Fear of compassion is thought to develop as an adaptive strategy when an individual has experienced harsh or punishing environments early in life or not experiencing safeness in early attachment relationships, and is also likely to impact on the ability to self‐soothe (Gilbert, 2005). The aim of the social BMAC is to shift attention to things that happen naturally in people's lives; to savour positive social interactions with the aim of decreasing perceived threat. However, for those high in self‐criticism or with a fear of compassion, it is possible that attempts to activate this affiliative system will be associated with threat. It is therefore important to test empirically how individuals respond to imagery of a real event and what might predict these responses.

The primary aim of the current study is to investigate individuals' emotional reactions to the BMAC technique. It was predicted that there will be a significant increase in feelings of safeness and warmth following the social BMAC. A reduction in active (drive‐related) positive affect was not, however, expected. It was also predicted that there would be a significant decrease in negative affect following the social BMAC. Finally, it was predicted that fear of compassion and self‐attacking (inadequate and hated) will be negatively associated with the degree of change in feelings of safeness and warmth following the social BMAC. The sensitivity to threat‐based emotions is an evolved tendency existing upon a continuum, and as such within a student sample we expect individual differences in this regard. This is therefore a suitable population to trial the social BMAC, in particular, establish what individual characteristics may influence its efficacy. Identifying such relationships in a student sample is a helpful first step before research into the social BMAC is extended to clinical groups.

## Method

### Participants

Participants from a University in the North of England were recruited via an online advert placed on the University announcement service. Participants who left their contact details were entered into a draw to win a £150 shopping voucher. Participants were included if they were aged over 18 years, were able to follow written and verbal instructions, and had access to a computer with headphones or speakers. Participants were not asked if they had a clinical diagnosis. Two‐hundred and thirty‐one participants started the study (151 female, *M*_age_ = 24.23 years, *SD* = 6.84), 155 completed all the pre‐task assessments, whilst 124 completed both pre‐ and post‐task assessments (77 female, *M*_age_ = 24.89 years, *SD* = 7.95). Mann–Whitney tests did not identify any significant differences between those who completed all pre‐ and post‐task study measures and those who did not (*n* = 32–74; *p *≥* *.10). Participants were given the option to leave their contact details to receive a link to a short follow‐up assessment 2 weeks later. Forty‐nine participants completed the follow‐up assessment (32 female, *M*_age_ = 24.39 years, *SD* = 6.09).

Scores on the study measures were similar to results obtained from other non‐clinical samples (Gilbert, McEwan, Matos, & Rivis, 2011; Gilbert *et al*., 2004, 2008) for relaxed affect (*M *=* *14.33 vs. 13.74 here); safe affect (*M *=* *11.07 vs. 10.82 here) fear of compassion‐self (*M *=* *16.12 vs. 13.40 here) fear of compassion from others (*M *=* *15.78 vs. 13.00 here) inadequate self‐attacking (*M *=* *16.75 vs. 19.09 here) hated self‐attacking (*M *=* *3.86 vs. 4.09 here), but the current sample had more notably lower levels of active positive affect (*M *=* *21.14 vs. 14.15 here), negative affect (Crawford & Henry, 2004; *M *=* *16.00 vs. 7.27 here) and fear of compassion for others (Gilbert *et al*., 2008; *M *=* *21.18 vs. 14.25 here). These results suggest on the whole a slightly healthier sample, and may represent location‐specific differences since these studies took place in different locations. Despite the apparently ‘healthier’ scores, there was variability here with some individuals' scores suggesting difficulties around affect and self‐compassion.

### Measures

#### State positive and negative affect

The Types of Positive Affect Scale (Gilbert *et al*., 2008) is an 18 item scale rated on a 5‐point Likert scale. The instructions were changed to ask participant how they were feeling at the moment (e.g., ‘secure’, ‘calm’, ‘active’) and the Likert scale was changed so rather than measuring trait emotions (0 = not at all like me, 4 = extremely like me) it measured state emotions (0 = not at all, 4 = extremely). The scale consists of three subscales; active (e.g., ‘Energetic’, ‘Lively’), relaxed (e.g., ‘peaceful’, ‘calm’) and safe/warm (e.g., ‘Content’, ‘secure’). The internal consistency in the current sample was between α = .86 and .94. The safe/warm subscale has been found to be the best predictor of alexithymia, mindfulness and depression when compared to the other subscales, supporting the distinction between types of positive affect (Gilbert *et al*., 2011). The negative subscale of the Positive and Negative Affect Scale (Watson, Clark, & Tellegen, 1988) was used to measure negative affect. This scale consists of 10 items rated on a 5‐point Likert scale and has previously demonstrated high reliability (Crawford & Henry, 2004). The internal consistency in the present sample was α = .89.

#### Social safeness

The Social Safeness and Pleasure Scale (SSPS; Gilbert *et al*., 2009) consists of 11 items rated on a 5‐point Likert scale. Instructions were modified to measure state social safeness and pleasure. Participants are asked how they feel ‘right now’ (e.g., ‘I feel connected to others’; 0 = agree, 4 = disagree). Kelly, Zuroff, *et al*. (2012) provide evidence that Social Safeness as operationally distinct from positive and negative affect and was more strongly related to various indicators of vulnerability and psychopathology. The internal consistency for the state measure of social safeness in the current sample was α = .95.

#### Self‐attacking

The Forms of Self‐Attacking and Self‐Reassurance Scale (FSCRS; Gilbert *et al*., 2004) is a 22 item scale rated on a 5‐point Likert scale (e.g., ‘I am easily disappointed with myself’). The scale comprises three subscales (inadequate‐self, hated‐self and reassure‐self). Kupeli, Chilcot, Schmidt, Campbell, and Troop (2013) confirmed the three sub‐scales as the best‐fitting structure in a confirmatory factor analysis and validity was supported by finding that the FSCRS was associated with depression. Only the inadequate‐self and hated‐self subscales are used in this study. The present study demonstrated an internal consistency of α > .87 for inadequate‐self and hated‐self subscales in the present sample.

#### Fear of compassion

The Fear of Compassion Scale (FCS; Gilbert *et al*., 2011) is a 38 item scale rated on a 4‐point Likert scale. The scale consists of three subscales; expressing compassion for others, responding to the expression of compassion from others, and expressing kindness and compassion towards oneself. Internal consistency on the subscales in the current sample was between α = .88 and α = .94. The FCS has demonstrated expected relationships with alexithymia, self‐criticism, depression, anxiety and stress in healthy samples, supporting its validity (Gilbert *et al*., 2011, 2012).

### Design

A within‐group repeated‐measures design was utilized, with participants completing measures of positive and negative affect at pre‐task, post‐task and at 2‐week follow‐up.

### Procedure

Ethical approval was granted by the University research ethics committee. An initial pilot study (*n *=* *10) was conducted in order to ensure that there were no technical difficulties with the online study and to get feedback on the quality of audio recording, the length of imagery exercise, and whether the instructions were clear. The general feedback was that the instructions were clear, the audio quality was good, the pauses in the imagery exercise were the right length, but that the imagery exercise was too long. In light of the feedback the relaxation component of the imagery exercise was shortened for the main study.

In the study proper, participants followed a link on the study advert to access the online study. Participants first completed general/trait self‐report measures of self‐attacking, fear of compassion, and social safeness/pleasure. They then completed state measures of social safeness/pleasure, and positive and negative affect. Participants were then asked to recall a recent positive memory of being with another person and to complete the social BMAC prompt sheet. Following this, participants followed auditory instructions, which guided them through an initial relaxation exercise and the social BMAC. The aim of the relaxation exercise was to focus individuals' attention to themselves and the present moment. The social BMAC guides the person through a positive social memory. Participants were encouraged to engage all the senses, think about the meaning of the memory to them, savour the positive feelings they experienced, and consider the positive feelings in the mind of another before reflecting upon the feelings they experience as well as what this means to them. It then asks the person to savour that feeling. The BMAC was presented using a male voice. We could identify no research concerning possible bias in affect related tasks related to the gender of presenter. The script for the social BMAC is provided in the Appendix. A copy of the recording is available from the first author. Participants were then asked to re‐complete state measures of positive and negative affect, and social safeness/pleasure. The study took approximately 45 min to complete.

Participants who chose to leave their contact details were contacted via email 2 weeks after completing the study and with a link to the follow‐up study. The follow‐up study involved repeating the state measures of positive and negative affect and social safeness and pleasure.

### Data analysis

An analysis of change in affect across the three time periods (pre‐, post‐task, follow‐up) was undertaken using non‐parametric Friedman's tests due to non‐normality in the affect variables. Bootstrapped paired *t*‐tests (5,000 re‐samples) to compared scores across two time points (pre vs. post). Bootstrapping provides a non‐parametric way of generating Confidence Intervals (CI) and so determining significance (Mooney & Duval, 1993). Analyses of predictors of change in affect were undertaken via multiple linear regression. As the goal of the BMAC was primarily to produce a change in safe/warm affect and social safeness we focussed on the predictors of change for these outcomes. The outcome was either safe/warm affect or social safeness at post‐task, with these variables at pre‐task (either safe/warm affect or social safeness depending on the outcome) entered as a covariate, so that the change in affect was being assessed. The different forms of self‐attacking (inadequate‐self, hated‐self) and FCSs were then entered as predictors of this change. CI for regression coefficients were bootstrapped with 5,000 re‐samples as residuals demonstrated a degree of heteroscedasticity. Multicolinearity was not present (Tolerance > 0.2). BootCI (Bakerman, 2014) was used to calculate bootstrapped (10,000 resamples) percentile CI for the change in *R* ^2^ associated with subsequent steps in the regression (Algina, Keselman, & Penfield, 2007).

## Results

### Emotional reactions to the social BMAC

Friedman's analysis of variance showed that there was a significant effect of time (pre, post and follow‐up) on relaxed positive affect (*p *≤* *.01), safe/warm positive affect (*p *≤* *.01), social safeness and pleasure (*p *≤* *.01), and negative affect (*p *≤* *.01). There was no significant effect of time on active positive affect (*p *=* *.58). These significant effects were followed‐up with bootstrapped pair‐wise, paired *t*‐tests, the results of which are reported in Table 1. It was found that safe/warm positive affect, relaxed positive affect and feelings of social safeness and pleasure significantly increased from pre‐task to post‐task (*p *≤* *.01) and that negative affect significantly decreased (*p *≤* *.01). There was no significant change in any of the measures of affect from pre‐task to follow‐up, suggesting that change in affect was momentary and not sustained over time.

**Table 1:** Change in affect following the Broad Minded Affective Coping

Measure	Time	*N*	Mean	*SD*	95% CI for mean difference		*d*
					Lower	Upper	
Active positive affect	T1	124	13.95	8.22	−1.00	0.91	.01
	T2		14.01	8.20			
	T1	49	14.37	8.39	−1.49	2.74	−.09
	T3		13.67	7.63			
Relaxed positive affect	T1	124	13.96	6.27	−3.60	−2.18	.48^a^
	T2		16.86	5.85			
	T1	49	13.55	6.71	−1.46	1.86	−.03
	T3		13.37	5.78			
Safe/warm positive affect	T1	124	10.88	3.62	−1.76	−1.02	.37^a^
	T2		12.26	3.67			
	T1	49	10.86	3.80	−0.47	1.18	−.11
	T3		10.47	3.29			
Negative affect	T1	123	7.71	7.46	2.23	4.41	−.47^a^
	T2		4.39	6.45			
	T1	49	8.27	7.74	−0.08	4.06	−.27
	T3		6.37	5.87			
Social safeness	T1	123	39.16	9.99	−3.28	−1.79	.25^a^
	T2		41.67	10.09			
	T1	49	40.35	9.83	−1.86	2.35	−.03
	T3		40.10	9.37			

Note

T1 = pre‐task; T2 = post‐task; T3 = follow‐up.

CI are bootstrapped, bias‐corrected and accelerated confidence intervals based on 5,000 re‐samples. Cohen's d for within‐group change based on formula from Borenstein, Hedges, Higgins, and Rothstein (2009).

a *p* ≤ .01.

### Predictors of responses to the social BMAC

#### Social safeness and pleasure

In the first multiple hierarchical regression analysis, post‐task social safeness score was entered as the outcome variable. The first step, including pre‐task social safeness, resulted in *f*(1, 121) = 561.52, *p *<* *.01, and *R* ^2^ = .82 (bootstrapped 95% CI: 0.749–0.881). Including the five predictors (hated‐self, inadequate‐self, fear of compassion subscales) made a small but significant improvement in the variance explained by this model, *f*(5, 116) = 2.01, *p *=* *.08, Δ*R* ^2^ = .02 (bootstrapped 95% CI: 0.006–0.047). This was significant at the α = .05 level for the bootstrapped *R* ^2^, though not with the traditional parametric test for change in *R* ^2^. The regression coefficients and associated CI for all variables in the regression are reported in Table 2. Notably, inadequate‐self was the only significant predictor of change in social safeness following the social BMAC, with greater inadequate‐self attacking leading to a greater improvement in social safeness, but this was a relatively small effect.

**Table 2:** Regression of post‐task social safeness and pleasure score on fear of compassion and self‐attack variables

Model		*B*	95% CI		β	*r*_sp_
			Lower	Upper		
1	Pre‐task SSPS	0.92^a^	0.84	0.99	.91	–
2	Pre‐task SSPS	0.93^a^	0.84	1.03	.92	.66
	Inadequate‐self	0.14^a^	0.02	0.25	.13	.09
	Hated‐self	−0.00	−0.27	0.31	−.00	−.00
	FCS‐expressing to others	−0.02	−0.13	0.09	−.01	−.01
	FCS‐receiving from others	−0.06	−0.19	0.07	−.06	−.04
	FCS‐self	−0.06	−0.17	0.06	−.07	−.05

Note

SSPS = Social Safeness and Pleasure Scale; FCS = Fear of Compassion Scale.

a *p* ≤ .05.

#### Safe/warm positive affect

The multiple hierarchical regression analysis was then repeated with post‐task safe/warm positive affect score being entered as the outcome variable. The first step, including pre‐task safe/warm affect, resulted in *f*(1, 122) = 283.79, *p *<* *.01, and *R* ^2^ = .70 (bootstrapped 95% CI: 0.592–0.790). Including the three predictors (hated‐self, inadequate‐self, fear of compassion) made a significant improvement in the variance explained by this model, *f*(5, 117) = 3.30, *p *<* *.01, Δ*R* ^2^ = .05 (bootstrapped 95% CI: 0.014–0.104). The regression coefficients and associated CI for all variables in the regression are reported in Table 3. Notably, inadequate‐self, hated‐self and fear of expressing compassion towards others significantly predicted change in safe/warm affect following the social BMAC, with inadequate‐self attacking leading greater improvement, whilst hated‐self attacking and fear of expressing compassion to others led to less improvement in reported safe/warm affect.

**Table 3:** Regression of post‐task safe/warm affect on fear of compassion and self‐attack variables

Model		*B*	95% CI		β	*r*_sp_
			Lower	Upper		
1	Pre‐task safe/warm	0.85^a^	0.73	0.96	.84	–
2	Pre‐task safe/warm	0.84^a^	0.69	0.97	.82	.71
	Inadequate‐self	0.08^a^	0.02	0.13	.19	.13
	Hated‐self	−0.18^a^	−0.31	−0.06	−.22	−.14
	FCS‐expressing to others	−0.06^a^	−0.12	−0.01	−.14	−.12
	FCS‐receiving from others	0.02	−0.03	0.07	.05	.03
	FCS‐self	−0.01	−0.06	0.04	−.03	−.02

Note

SSPS = Social Safeness and Pleasure Scale; FCS = Fear of Compassion Scale.

a *p* ≤ .05.

## Discussion

The primary aim of this study was to investigate individuals' emotional reactions to the mental imagery of a positive social memory using the BMAC technique. It was predicted that the social BMAC would be associated with improvement in feelings of safeness and warmth, whilst sadness would decline, and that fear of compassion and self‐attacking (inadequate and hated) would negatively predict improvement in safeness and warmth. As predicted, the results demonstrated significant, small‐to‐moderate increases in safe/warm positive affect and relaxed positive affect and small but significant increases in feelings of social safeness and pleasure following the social BMAC. There was also a significant decrease in negative affect. These changes were momentary and not sustained in the 2‐week follow‐up period. This is perhaps not surprising considering the brief nature of the task. Future research looking at more sustained or repeated use of the social BMAC would be helpful in determining if longer term benefits can be identified. There was no significant change in active positive affect, which is consistent with the theory that the social BMAC would tend to operate on specific forms of positive affect. Caution is of course needed in interpreting these findings as the lack of a randomized control group means the observed change cannot definitely be attributed to the task. Future studies adopting control groups are therefore needed. Nonetheless, these results provide preliminary evidence for the efficacy of the social BMAC.

Gilbert's (2005) three systems affect regulation model would suggest that the affiliative system can dampen down the threat and drive system and allow for feelings of social safeness. The differential stimulation of positive affect systems supports Gilbert's three systems theory (Gilbert, 2005, 2009), in that the social BMAC appears to activate the affiliative system, resulting in momentary increases in safe/warm and relaxed feelings, which increases feelings of social safeness and decreases negative emotions related to threat. These findings are also consistent with previous research that suggests that social safeness is an emotional response to affiliation (Kelly, Zuroff, *et al*., 2012). The results could also be understood in the context of a cognitive model (Beck, 1976); whereby a positive interpretation of an event (the memory) leads to the experience of positive emotions. The suggestion that positive emotions broaden attention and reduce focus on threat may also explain findings (Fredrickson, 2001).

In contrast to predictions, inadequate self‐attacking positively predicted greater improvement in both safe/warm positive affect and social safeness/pleasure. It was originally predicted that high levels of self‐attacking (both forms) develop as a consequence of an inability to experience affiliative affect. Inadequate self‐attacking is thought to be for self‐correction, whereas the function of hated self‐attacking is thought to be for self‐punishment (Gilbert *et al*., 2004). Considering the self‐corrective function of inadequate self‐attacking (Gilbert *et al*., 2004; Gilbert & Irons, 2005), individuals high in this variable may be seen as striving to achieve and to get things right in order to gain approval from others. It may be that these individuals engage well with the BMAC because they are striving to do well. The social BMAC encourages the person to focus on and savour the positive feeling another has in relation to oneself. It could also be that individuals high in inadequate self‐attacking are receptive to receiving positive regard from others, even if they struggle to generate this by themselves. As the social BMAC encourages the person to focus on and savour the positive feelings they receive from others these individuals high in inadequate self‐attacking may still benefit from the task.

In contrast, individuals who self‐attack in order to punish themselves (hated‐self) and who fear expressing compassion towards others experienced less safe/warm affect in response to the task. Both these variables may leave individuals less receptive to signals of positive regard from others, possibly because they have developed a threat system that dampens down affiliative emotions in order to prevent external attack (Gilbert, 2000). These variables did not affect the experience of social safeness. Thus, such individuals may still be able to savour and enjoy memories of social connectedness but struggle more to generate a secure and safe affective state. Notably, because these three variables (hated‐self, inadequate‐self, fear of compassion) are positively inter‐correlated but act in different ways upon the outcome, there is a suppression effect at play in this analysis (Paulhus, Robins, Trzesniewski, & Tracy, 2004). As such it may be important for future research to consider these variables together within statistical models in order to account for such suppression effects.

It should, however, be noted that these effects were relatively small, explaining only a small amount of variance in the outcomes. Thus, whilst response to the social BMAC does appear to vary as a function of these variables, the impact they have, at least in terms of response to a single BMAC exercise, is still minimal. It may be that with repeated use of the BMAC, as part of a longer term intervention, the effect that self‐attacking styles and fear of compassion have on change would be more pronounced.

Taken together, these findings could have numerous clinical implications. Tarrier (2010) has suggested that the BMAC could be used in cognitive therapy with different aims such as providing a temporary lift in mood which could increase motivation to engage in activities, improving mood before/after exposure work, and helping to build positive schema. The social BMAC significantly increased positive affect and feelings of safeness in this group, although the degree of improvement was less in individuals with more hated‐self attacking and fear of compassion. Clinically, these findings are consistent with Gilbert's tripartite model of emotional systems (Gilbert, 2005, 2009), which provides a basis for formulating a person's likely response to affiliative affect and critically, how previous experiences may have led this type of emotion to be conditioned to threat‐based emotions. Before stimulating affiliative affect through use of interventions such as the social BMAC, it may therefore be helpful for clinicians to use the FCS (Gilbert *et al*., 2011) and the forms of self‐attacking and self‐reassurance scale (Gilbert *et al*., 2004) to aid them in this formulation and to decide which intervention would be most appropriate.

A novel aspect of this study was the delivery of the intervention via the internet. The results provide proof‐of‐concept evidence for the possibility of delivering an imagery‐based intervention in this way. The idea of internet‐mediated psychological interventions has become an area of increasing clinical and research interest (see review by Barak, Hen, Boniel‐Nissim, & Shapira, 2008). The potential for improving the accessibility of interventions, and bring interventions out of the therapy room and into individual's everyday lives are some of the potential benefits of internet‐mediated intervention. Some have further suggested the potential for online or mobile phone‐based interventions to become individualized to a particular clients need (e.g., adjusting the timing or form of interventions delivered; Kelly, Gooding, *et al.,* 2012). The social BMAC represents one specific intervention that could be readily applied via the internet, or incorporated into a mobile phone application.

A limitation of this study is that a comparison group was not used. It is therefore unclear whether it was the social BMAC that brought about change or whether it was some non‐specific element of the intervention. It could be that focusing on any sort of imagery could bring about change in affect. In future research, it would be helpful to have a comparison group who complete a relaxation imagery exercise to allow for the exploration of change brought about by the social BMAC above that of relaxation alone. It would also be useful to evaluate this technique within a clinical group, where problems around accessing positive social memories and emotions will be more pronounced. As self‐criticism is a trans‐diagnostic phenomenon (Gilbert & Irons, 2005), this could be carried out with various clinical populations such as those with depression, anxiety, borderline personality disorder, bipolar disorder, deliberate self‐harm, or psychosis. Nonetheless, the current study helps us to establish a proof‐of‐concept regarding the social BMAC.

## Social Broad Minded Affective Coping script

- Keep your eyes closed and allow your attention to move to the positive experience.
- Where were you?
  ○ Inside or outside?
  ○ Try and focus on what you can see
  ○ Move around the memory – build the scene in your mind
    - If you were outside, what was the weather like, what could you see?
    - If you were inside, think about the floor, walls, furniture.
    - Focus on each thing you can see around you.
- Now think about the other person or people in the memory.
  ○ Focus on their face
  ○ What was their expression?
    - Look at their eyes – the colour
    - Look at their nose
    - Look at their mouth
  ○ What were they wearing?
    - Focus on their clothes and the colour
  ○ What were they doing?
    - Recreate the image of what they were doing in your mind.
- Now try and focus on what you could hear. Allow the sounds to fill your mind.
  ○ Try and focus on the other person's voice, the tone.
    - What did they say? Try and recreate the sound of the words.
  ○ Think about other sounds in the environment.
    - Think through each sound and allow it to fill your mind
- Now try and focus on the smells in the memory.
  ○ Was there any food or drinks?
  ○ Perfumes or aftershaves?
  ○ If you are outside, are there any other smells like fresh grass
- Now try and focus on any taste in this memory
  ○ Did you eat or drink anything?
    - Really savour the taste of this, allow the memory to fill your mind.
- Now try and focus on the feel of things in the memory
  ○ Did you touch anything or anyone?
  ○ How did it feel?
- You are free to move around this image at you own will.
- If there is a strong part of the image, return to this if it begins to fade – think of word to bring you back here at any point.
- Focus now on the strongest and most positive bit of this memory.
  ○ How did it make you feel?
    - Allow the feeling to wash over you, to fill your mind.
    - Really savour this feeling.
- Think of a word to bring you back at any point.
- Think about what the memory means to you.
- What went through your mind?
- Why was it important to you?
- Think of a word to bring you back at any point.
- How did the other person or people in the memory feel?
- How does that make you feel – that they feel like this?
- What does this mean to you – that other people think or feel this way about you?
- What does this memory mean about your life?
- How can this memory help?
- How does it show your positive qualities? Think about these qualities.
- Once again, think about the feeling and allow it to fill your mind. You can go back to this at any time. Think of a work that could bring you back here any time you want to.
- Now just begin to become aware of the room you are in.
- When you are ready open your eyes.
